# LncRNA *SNHG1* and RNA binding protein *hnRNPL* form a complex and coregulate *CDH1* to boost the growth and metastasis of prostate cancer

**DOI:** 10.1038/s41419-021-03413-4

**Published:** 2021-02-01

**Authors:** Xiao Tan, Wen-bin Chen, Dao-jun Lv, Tao-wei Yang, Kai-hui Wu, Li-bin Zou, Junqi Luo, Xu-min Zhou, Guo-chang Liu, Fang-peng Shu, Xiang-ming Mao

**Affiliations:** 1 Department of Urology, The Affiliated Hospital of Southwest Medical University, Luzhou, Sichuan China; 2 Department of Urology, Zhujiang Hospital, Southern Medical University, Guangzhou, Guangdong China; 3 Department of Urology, Minimally Invasive Surgery Center, the First Affiliated Hospital of Guangzhou Medical University, Guangdong Key Laboratory of Urology, Guangzhou Institute of Urology, Guangzhou, Guangdong China; 4 Department of Urology, Guangzhou Women and Children’s Medical Center, Guangzhou Medical University, Guangzhou, Guangdong China

**Keywords:** Metastasis, Prostate cancer

## Abstract

The interaction between LncRNA and RNA-binding protein (RBPs) plays an essential role in the regulation over the malignant progression of tumors. Previous studies on the mechanism of *SNHG1*, an emerging lncRNA, have primarily focused on the competing endogenous RNA (ceRNA) mechanism. Nevertheless, the underlying mechanism between *SNHG1* and RBPs in tumors remains to be explored, especially in prostate cancer (PCa). *SNHG1* expression profiles in PCa were determined through the analysis of TCGA data and tissue microarray at the RNA level. Gain- and loss-of-function experiments were performed to investigate the biological role of *SNHG1* in PCa initiation and progression. RNA-seq, immunoblotting, RNA pull-down and RNA immunoprecipitation analyses were utilized to clarify potential pathways with which *SNHG1* might be involved. Finally, rescue experiments were carried out to further confirm this mechanism. We found that *SNHG1* was dominantly expressed in the nuclei of PCa cells and significantly upregulated in PCa patients. The higher expression level of *SNHG1* was dramatically correlated with tumor metastasis and patient survival. Functionally, overexpression of *SNHG1* in PCa cells induced epithelial–mesenchymal transition (EMT), accompanied by down-regulation of the epithelial marker, *E-cadherin*, and up-regulation of the mesenchymal marker, *vimentin*. Increased proliferation and migration, as well as accelerated xenograft tumor growth, were observed in *SNHG1*-overexpressing PCa cells, while opposite effects were achieved in *SNHG1*-silenced cells. Mechanistically, *SNHG1* competitively interacted with *hnRNPL* to impair the translation of protein *E-cadherin*, thus activating the effect of *SNHG1* on the EMT pathway, eventually promoting the metastasis of PCa. Our findings demonstrate that *SNHG1* is a positive regulator of EMT activation through the *SNHG1*-*hnRNPL*-CDH1 axis. *SNHG1* may serve as a novel potential therapeutic target for PCa.

## Introduction

Prostate cancer (PCa) is the frequency life-threatening tumor in male genitourinary system^1^. The malignant transformation of the prostate follows a multi-step process, starting with prostatic intraepithelial neoplasia, Then there is localized prostate cancer, followed by locally invasive advanced prostatic adenocarcinoma, which eventually develops into metastatic PCa^2^. The patient eventually developed metastatic castration resistant prostate cancer due to castration resistance and metastasis, resulting in death. There’s growing evidence that epithelial–mesenchymal transition (EMT) emerges a great role in the metastasis of various cancers, including PCa^3–5^. The progression of PCa is closely related to the proliferation and invasion phenotype of cancer cell^6^. However, the cellular and molecular mechanisms responsible for the metastasis PCa are incompletely understood.

Long non-coding RNAs (LncRNAs) are RNAs with a length of more than 200 nucleotides without protein-coding function. In recent years, the expansion of knowledge has revealed that lncRNAs play an important role in multiple biological processes, such as alternative splicing, nuclear import, imprinting, cell differentiation and RNA decay^7^. The abnormal expression of lncRNAs have also been reported to contribute to tumorigenic processes of many human malignancies, including PCa^8,9^. In PCa, recent studies have revealed that LncRNA DLX6-AS1 enhances PCa Malignant Phenotype and Lymph Node Metastasis^10^, and LncRNA AC245100.4 promotes the proliferation of PCa through binding to HSP90^11^. Small nucleolar RNA host gene 1(*SNHG1*), a novel LncRNA, has shown to be aberrantly high expression and oncogenic characteristics in various cancers^12^. Previous studies have illustrated that *SNHG1* was upregulated in PCa and was associated with PCa proliferation through the namely competing endogenous RNA (ceRNA) mechanism^13,14^. While, the specific function of *SNHG1* have not been well investigated in the context of PCa metastasis and the underlying mechanism are also needed to be elucidated.

In this study, we sought to determine the expression and the biological function of *SNHG1* in PCa, especially its role in metastasis. Expression levels of *SNHG1* were determined in the PCa primary tumor tissues and its correlations with clinicopathological parameters were also analyzed. We further investigated the effects of *SNHG1* on the aggressive phenotypes of PCa cell lines in vitro and in vivo. The regulatory role of *SNHG1* on *CDH1* were also explored to elucidate the potential mechanisms. Taken together, our results have demonstrated that *SNHG1* played a critical role in the progression of PCa.

## Results

### LncRNA *SNHG1* is upregulated in PCa tissues and sub-located in the nucleus

**Fig. 1:**
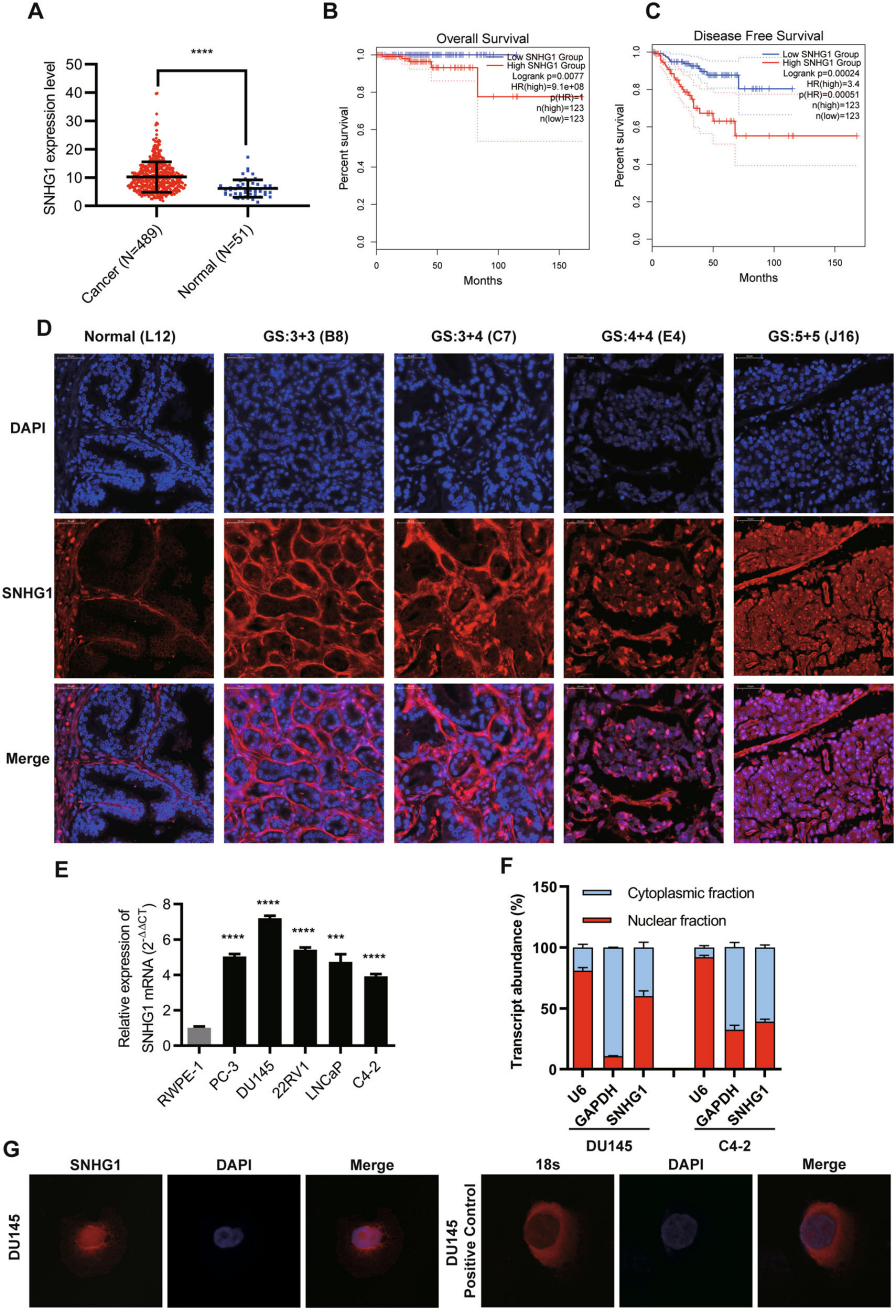
LncRNA *SNHG1* is overexpressed in prostate cancer tissues. **A** *SNHG1* is overexpressed in TCGA PCa data. **B**, **C** Survival analysis of *SNHG1* (TCGA PRAD) showed that both overall and disease-free survival were worse in the highly expressed group. **D** FISH analysis of PCa tissues confirmed that *SNHG1* was highly expressed in PCa tissues and located in the nucleus (G means Gleason grade group). **E** *SNHG1* expression was analyzed by qRT-PCR in five PCa cancer cell lines (DU145, PC-3, 22RV1, LNCaP, and C4-2), compared with the human Prostatic immortalized epithelial cells (RWPE-1). **F** Distribution of *SNHG1* in PCa cells detected by fractionation of DU145 and C4-2 cells followed by qRT-PCR. **G** FISH analysis of DU145 cell. The nuclei were stained with DAPI and 18 S was used as a positive control for cytoplasmic staining. GS means Gleason Score. Scale bar, 20 μm. All data are shown as the mean ± SD. *P < 0.05, **P < 0.01, ***P < 0.001 and ****P < 0.0001 by two-tailed Student’s *t* test.

To identify the expression and localization of *SNHG1* in PCa, we utilized bioinformatics to analyze the expression and prognosis of *SNHG1* in PRAD in TCGA database. As shown in (Fig. 1A–C), we identified *SNHG1* transcript level is overexpression in prostate cancer. Meanwhile, we evaluated the correlation between *SNHG1* level and clinical outcomes from TCGA database using Kaplan–Meier analysis and log-rank tests. In the PRAD group, the prognosis of 247 PCa patients with high transcriptional level of *SNHG1* was significantly worse than that of 248 patients with low expression of *SNHG1*.

Next, the expression and localization of *SNHG1* was validated in 67 PCa tissues and 14 adjacent normal tissues *via* RNA FISH. we divided the sample into high (++, +++) and low (−, +) *SNHG1* expression group according to the positive cell nuclear stain percentage of *SNHG1* and then explored the correlation between the clinicopathological characters of prostate cancer patients and *SNHG1* expressions. *SNHG1* was significantly overexpressed in 67.16% (45 of 67 PCa tissues) and low expression in all (14 of 14 normal adjacent tissues) normal samples and 32.84% (22 of 67) PCa tissues (Table 1 and Fig. 1D). Besides, as shown in Table 1, *SNHG1* transcript level was positively associated with Gleason Score but it may not be related to the patient’s age and clinical TNM stage in our study by Chi-square tests.

**Table 1:** Expression of *SNHG1* in normal prostate tissues and prostate cancer tissues.

	Group	N	SNHG1 expression		*χ*^2^	*p* value
			low	high		
Type	Normal	14	14	0	21.157	<0.001
	Adenocarcinoma	67	22	45		
Age	≤ 65	26	8	18	0.082	0.774
	> 65	41	14	27		
Clinical stage	I-II	42	15	27	0.423	0.516
	III-IV	25	7	18		
Primary tumor	T1-T2	44	16	28	0.723	0.395
	T3-T4	23	6	17		
Gleason score	≤ 6	17	9	8	4.175	0.041
	≥ 7	50	13	37		

*SNHG1* expression was determined by FISH; *p* value is from χ^2^-test -test.

A remarkably increasing frequency of positive expression of *SNHG1* was detected in prostate cancer specimens compared to normal prostate tissues (*P* < 0.001, χ^2^-test).

Furthermore, we validated the higher expression level of *SNHG1* in DU145, PC3, LNCaP, c4-2, and other PCa cell lines compared to that in RWPE-1 cell line (Fig. 1E). Then, we cultured PCa cell DU145 and C4-2 as cell models of high and low *SNHG1* expression in PCa. Through cellular fractionation assays (Fig. 1F) and RNA fluorescence in situ hybridization (FISH) (Fig. 1G), we demonstrated that *SNHG1* was mainly distributed in the nucleus of DU145 cells and cytoplasm of C4-2 cells. Taken together, our data confirmed that *SNHG1* is highly expressed in PCa tissues and cell lines, which might potentially serve as a novel independent predictor of overall survival in PCa.

### LncRNA *SNHG1* is essential for promoting PCa cells proliferation, migration in Vitro

**Fig. 2:**
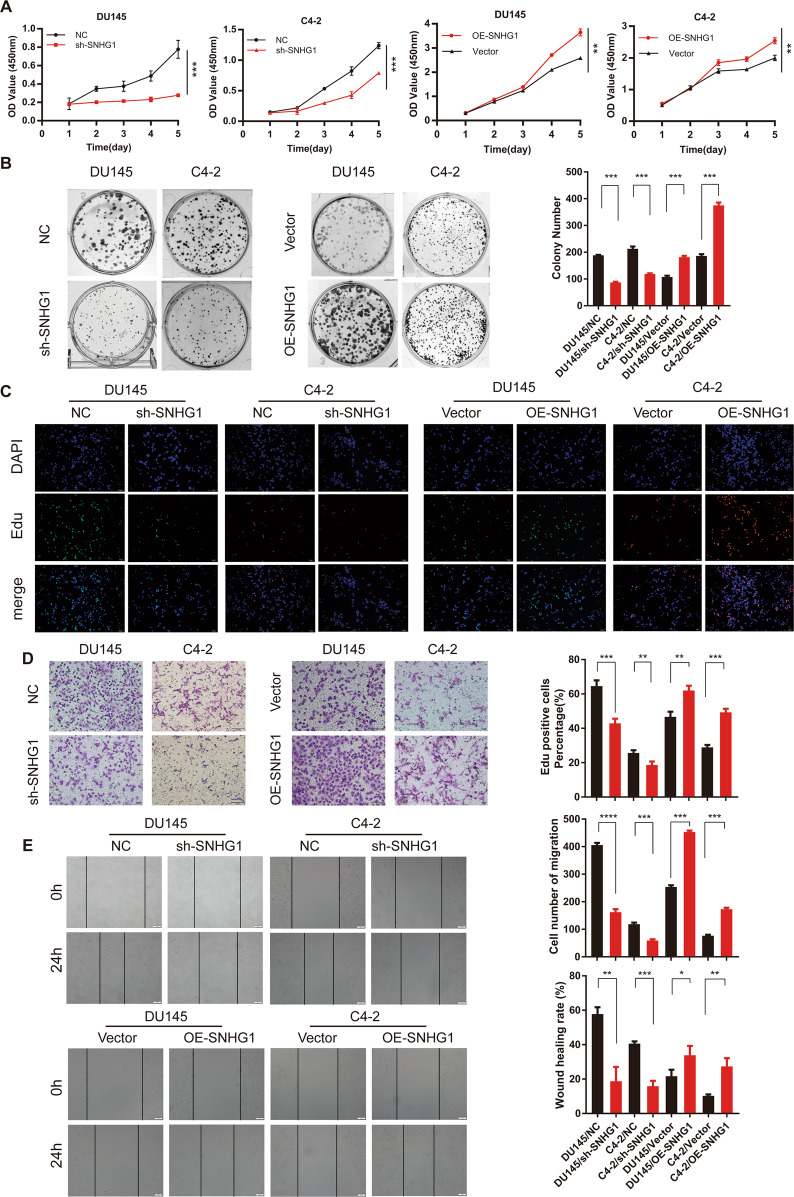
LncRNA *SNHG1* promotes PCa cell proliferation and migration in vitro. **A, B** CCK-8 assay and colony-formation assay were used to determine the proliferation ability of PCa cells by knocking down or overexpressing LncRNA *SNHG1*. **C** EdU assays were used to determine the proliferation ability of PCa cells. **D** Transwell assays showed that *SNHG1* knockdown or overexpression could inhibit or promote PCa cell migration. **E** *SNHG1* knockdown attenuated cell migration in DU145 and C4-2 cells, while up-regulation of *SNHG1* showed the opposite results. *P < 0.01, **P < 0.01, ***P < 0.001, ****P < 0.0001.

To figure out the potential role of *SNHG1* in promoting prostate cancer progression, siRNA or lentivirus shRNA were used to knock down the endogenous expression of *SNHG1* in DU145 and C4-2 cells and used an overexpression plasmid system to upregulate *SNHG1* in DU145 and C4-2 cells (Fig. S1A). CCK-8 assays, colonies formation and EDU assays demonstrated that upregulated *SNHG1* remarkably enhanced PCa cells proliferation, while *SNHG1* knockdown significantly blocked the proliferative abilities of PCa cells (Fig. 2A–C). Notably, *SNHG1* knockdown impaired the migration ability of PCa cells, while upregulated *SNHG1* transcription level promoted cell migration (Fig. 2D–E). These results suggested that *SNHG1* promotes cell proliferation, migration in PCa cells. Besides, SNHG1 overexpression did not affect the migration abilities of the immortalized prostatic epithelial cell line RWPE-1 cells (Fig. S1B–E).

### Knockdown LncRNA *SNHG1* inhibited PCa cells tumorigenesis in vivo

**Fig. 3:**
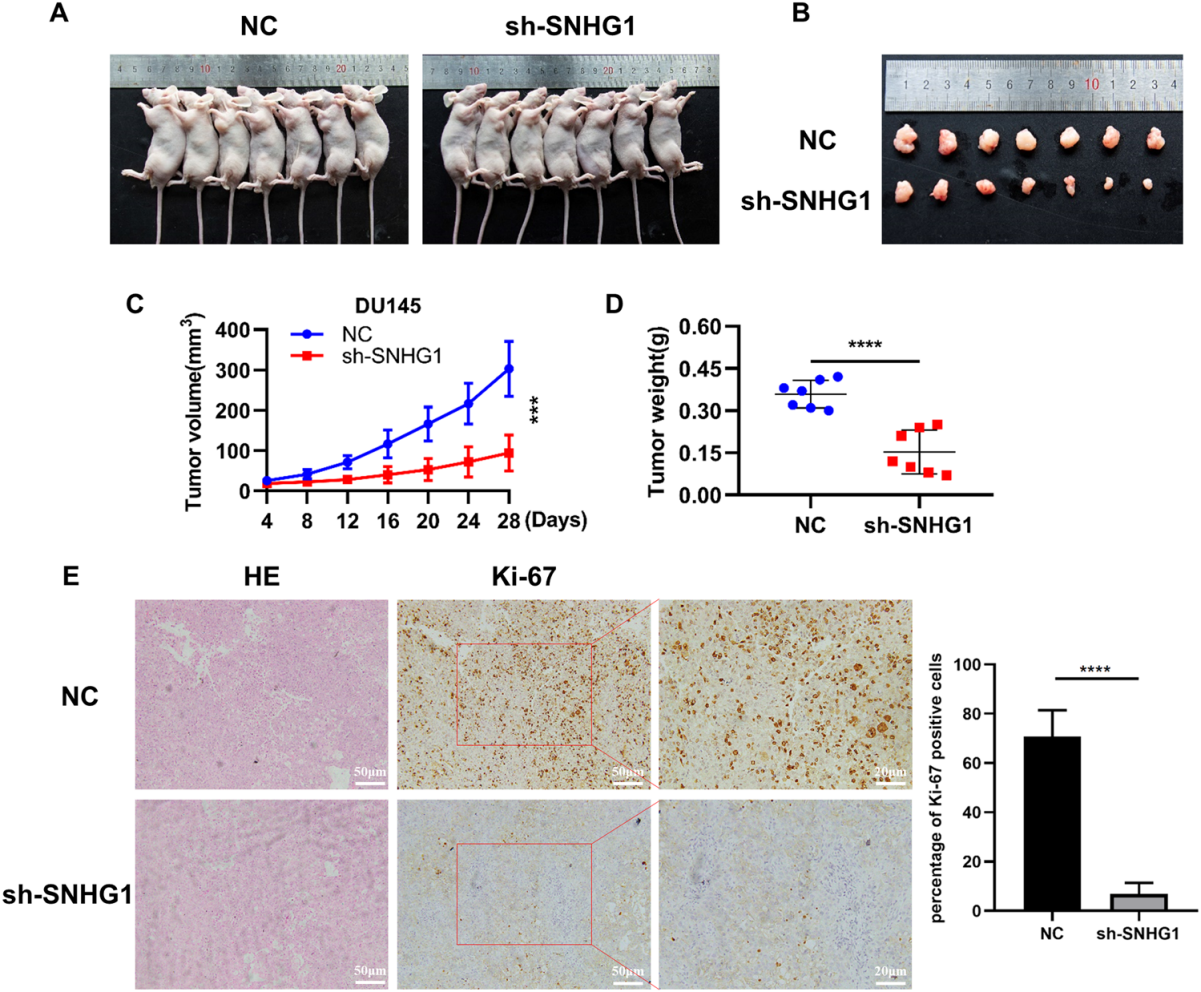
Subcutaneous tumor formation in nude mice confirmed that silencing *SNHG1* inhibited the proliferation of PCa cells in vivo. **A**, **B** Negative control or sh-*SNHG1*was stably transfected into DU145 cells, which were injected into the nude mice. **C** Tumor volumes were calculated after injection every 4 days. **D** Tumor weights were represented as means of tumor weights ± SD. **E** IHC analyses of ki-67 confirmed that the proliferation of subcutaneous tumor was inhibited in sh-*SNHG1* group. ***P < 0.001, ****P < 0.0001.

For the purpose of validating the potential impact of *SNHG1* depletion on the tumorigenesis of PCa in vivo, DU145 cells transfected with sh-*SNHG1* or Negative Control lentivirus vector were injected subcutaneously in male nude mice. Tumors in mice implanted in DU145/sh-*SNHG1* cells were remarkably smaller than those in the control cells (Fig. 3A, B). Likewise, *SNHG1* knockdown cells grew slower in compared with the control cohort, the average tumor volumes and weight in the ultimate experiment were significantly lower in the DU145/sh-*SNHG1* cohort versus the negative control lentivirus vector cohort (Fig. 3C, D). IHC staining of ki-67 antigen confirmed that subcutaneous tumor proliferation was significantly reduced in the sh-*SNHG1* group (Fig. 3E).

### Related genes of lncRNA *SNHG1* in PCa cells

**Fig. 4:**
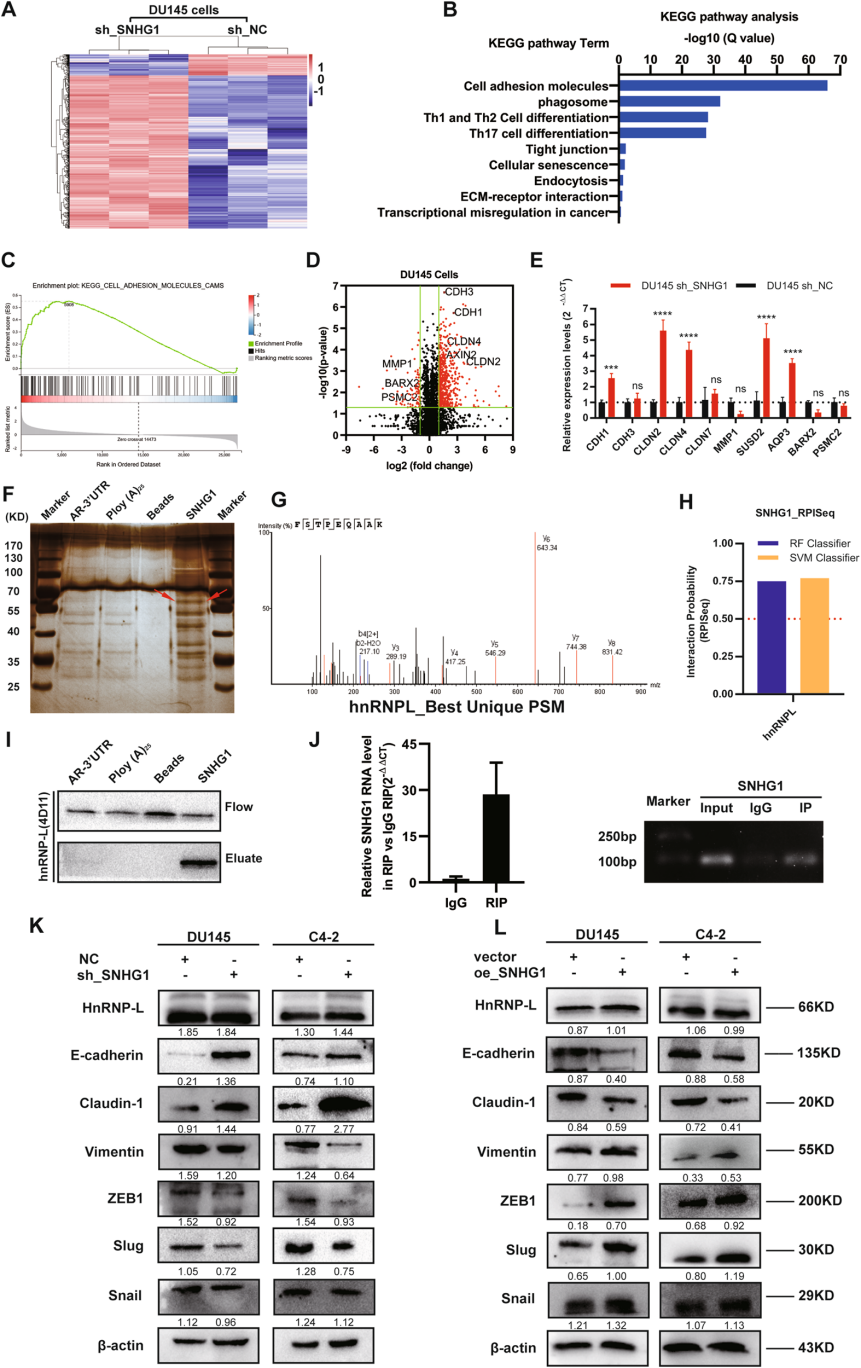
*SNHG1* could directly bind to *hnRNPL* and regulate EMT. **A** Hierarchical clustering of 1045 transcripts altered (≥1.5-fold change) in NC-treated cells and shRNA *SNHG1*-treated cells with three repeats. **B, C** KEGG analysis and differential genes GSEA enrichment analysis demonstrated that cell adhesion molecules are the potential targets of *SNHG1* pathway. **D** Volcanic map analysis of differential genes. **E** The altered mRNA levels of genes were selectively confirmed by qRT-PCR in knockdown *SNHG1*. **F** SDS-PAGE silver staining of RNA pull-down protein samples showed a significant difference in protein band from 55 to 70 kDa. **G** Differential protein band mass spectrometry showed that the protein was *hnRNPL*. **H** The interaction possibilities of *hnRNPL* and *SNHG1* were detected in RPIseq, and the results showed that *hnRNPL* could well bind with *SNHG1* well (RPISeq). **I** Pull-down assays showed that biotinylated *SNHG1* could retrieve *hnRNPL* in DU145 cells by western blot(flow-through was a input control). **J** RNA immunoprecipitation revealed that *hnRNPL* could also specifically bind to *SNHG1*. **K**, **L** The western blotting results after *SNHG1* knockdown in DU145 and C4-2 cells showed that the expression level of *hnRNPL* did not change, while E-cad and Claudin-1 were significantly up-regulated, Vimentin, Slug and ZEB1 were significantly decreased. overexpression of *SNHG1* showed an opposite effect of these protein in in DU145 and C4-2 cells. Error bars indicate means ± SD. ***P < 0.001, ****P < 0.0001.

To validate the potential related genes that may be regulated by *SNHG1* in PCa cells, RNA transcriptome sequencing was carried out in DU145/NC or DU145/sh-*SNHG1* cells. As a result of silencing *SNHG1*, a total of 837 genes greater than 1.5-fold change increased, whereas 208 genes exhibited a decrease in abundance (<−1.5 fold change) (Fig. 4A). Meanwhile, A thorough study of the oncology analysis highlighted the most obvious biological phenomena of overexpression associated with the pathways in cell adhesion molecules (CAMs) (Fig. 4B–D), and the RNA sequencing data were verified by qRT-PCR (Fig. 4E). However, the mechanism by which *SNHG1* regulates CAMs is still unclear.

### *SNHG1* activates the EMT pathway in PCa cells through interaction with *hnRNPL*

In order to further clarify the pathway of *SNHG1* regulating network, we used RNA-protein pull-down to analyze the protein molecules interacting with *SNHG1*. By silver staining of pull-down protein SDS-PAGE gel, we found that *SNHG1* was specifically bound to 60-70kda protein bands (Fig. 4F), the main protein of *SNHG1* interaction was identified as *hnRNPL* by mass spectrometry and western blot (Fig. 4G–I). Other potential interactors of SNHG1 were shown in Table S1. Besides, the interaction between *hnRNPL* and *SNHG1* was further predicted by RPISeq (Fig. 4H), and the binding relationship was double checked by *hnRNPL* RIP-PCR (Fig. 4J). Furthermore, western blot showed that *SNHG1* knockdown and overexpression did not affect the *hnRNPL* protein level, and it can regulate EMT pathway (Fig. 4K–L). Finally, our previous work confirmed that *SNHG1* triggers EMT pathway through directly binding to *hnRNPL* in the development of prostate cancer.

### *SNHG1*/*hnRNPL* complex regulate *E-cadherin* expression

**Fig. 5:**
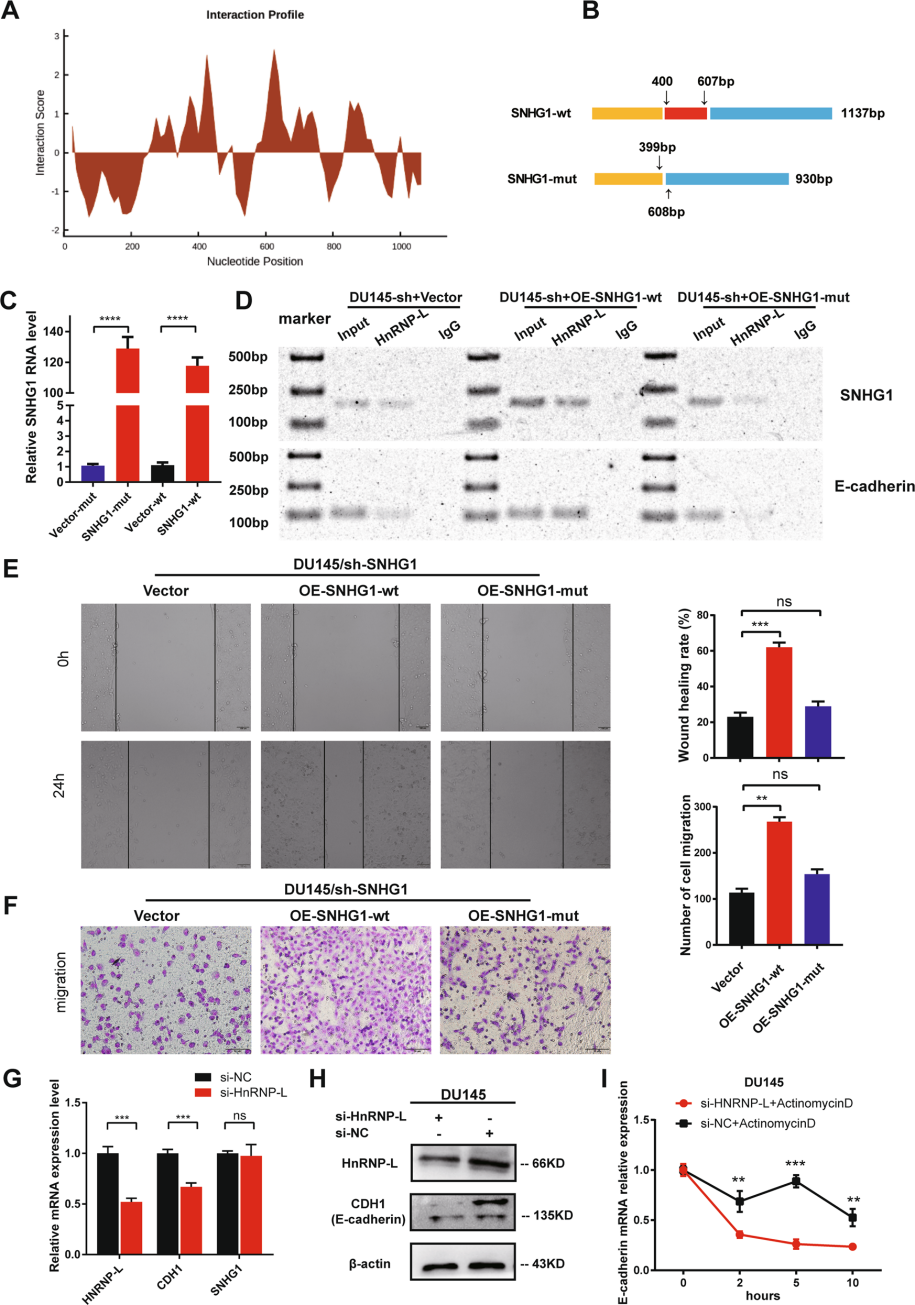
*SNHG1*/*hnRNPL* complex plays a vital role in PCa metastasis via stabilizing CDH1 mRNA. **A** CatRAPID fragment prediction revealed that the sequence of *SNHG1* at 400–600 bp had a significant binding possibility with *hnRNPL* (http://service.tartaglialab.com/page/catra-pid_group). **B** Schematic diagram of 400–607 bp truncated mutation in *SNHG1*. **C** Relative quantitative detection of truncated mutation and wild-type *SNHG1* overexpression by qRT-PCR. **D** *HnRNPL* RIP confirmed that knockdown *SNHG1* reduced the binding ability of *hnRNPL* to CDH1, and the overexpression of wild-type *SNHG1* could restore the binding ability, while the *SNHG1*-mut with poor binding ability of *hnRNPL* could not rescue *hnRNPL* to capture CDH1. **E**, **F** Effects of overexpression of wild type *SNHG1* or mutant *SNHG1* on cell migration by wound healing and migration assays. **G, H** Knockdown of *hnRNPL* suppressed the mRNA and protein expression of CDH1(E-cadherin). **I** *HnRNPL* knockdown in DU145 cells downregulated CDH1(E-cadherin) mRNA abundance, while ActinomycinD (2.0 μg/ml) were used to inhibit RNA synthesis. All data are shown as the mean ± SD. **P < 0.01, ***P < 0.001, ****P < 0.0001 by two-tailed Student’s *t* test.

Next, to investigate the exact binding site between *SNHG1* and *hnRNPL*, CatRAPID fragment prediction was used to reveal that the sequence of *SNHG1* at 400–600 bp have a significant binding possibility with *hnRNPL* (Fig. 5A). Then, we constructed *SNHG1*-mut truncated mutant overexpression vector with 400–607 bp deletion of *SNHG1* sequence compared with the *SNHG1*-wt (Fig. 5B). In DU145/sh-*SNHG1* cells, Vector, *SNHG1*-mut and *SNHG1*-wt plasmids were transfected, and the over-expression efficiency of plasmids was verified by qRT-PCR (Fig. 5C). Meanwhile, the results of *hnRNPL* RIP-PCR in aforesaid cells confirmed that 400–607 bp truncated *SNHG1*-mut could not bind to *hnNRPL* protein. In addition, *hnRNPL* also lost the ability to capture CDH1 to a great extent when it could not specifically bind to *SNHG1* compared with *SNHG1*-wt overexpressed cell lines (Fig. 5D). Meanwhile, Transwell and wound healing assays demonstrated that overexpressing mutant SNGH1 could not promote cell migration ability in PCa cells, when compared with the wild type *SNHG1* (Fig. 5E–F). As *hnRNPL* is essential for mRNA stability, we wondered if the *SNHG1*/*hnRNPL* stabilizes certain unknown downstream effector proteins, such as E-cadherin. Therefore, using siRNA targeting on *hnRNPL*, we effectively inhibited its expression in DU145 cells. Surprisingly, we found that E-cadherin was remarkably downregulated both in mRNA and protein levels, while *SNHG1* did not show obvious difference (Fig. 5G–H). We further found that knockdown of *hnRNPL* significantly reduced the mRNA stability of *CDH1(E-cadherin)* (Fig. 5I). Therefore, we demonstrated that SNHG1/hnRNPL complex could break the balance between *hnRNPL* and E*-cadherin* mRNA, causing to E-cadherin protein downregulation.

### *HnRNPL* knockdown reverses the carcinogenic effects of *SNHG1* promotion on cell metastasis

**Fig. 6:**
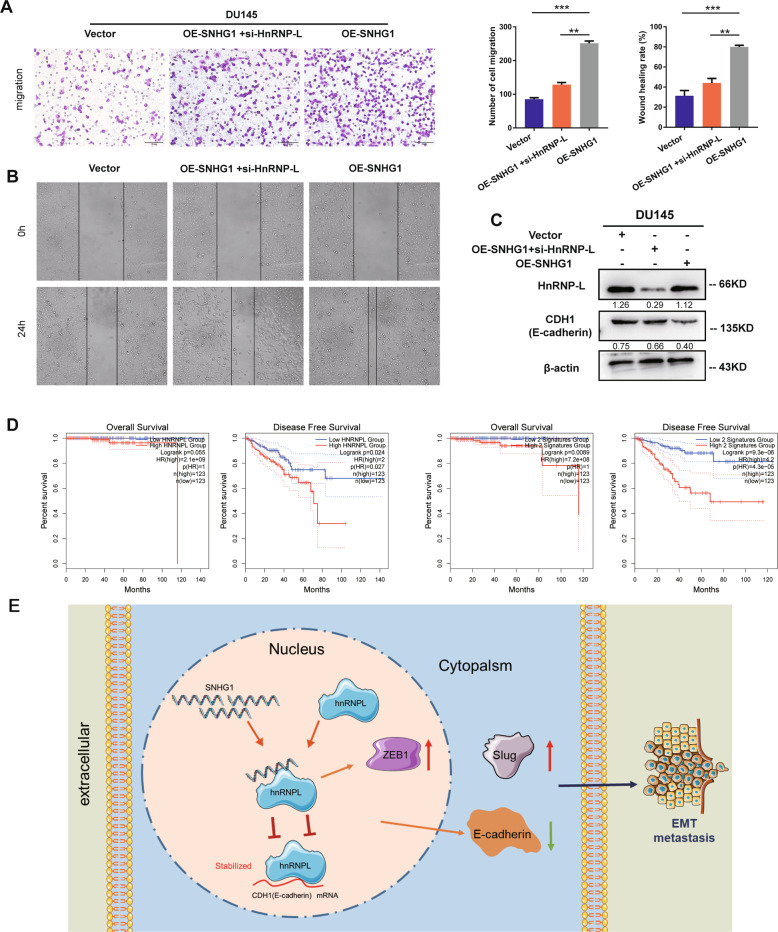
*HnRNPL* knocdown reverses the carcinogenic effects of *SNHG1* promotion on cell metastasis. **A**, **B** *HnRNPL* knockdown reversed the *SNHG1* overexpression-induced cell migration ability in PCa cells. **C** western blot showed that forced expression of *hnRNPL* reversed the *SNHG1* upregulating-induced inhibition of E-cadherin protein. **D** Survival analysis revealed that PCa patients with both low *SNHG1* and *hnRNPL* expression levels had better overall survival and disease-free survival (GEPIA, http://gepia.cancer-pku.cn/). **E** Schematic diagram displaying the mechanism underlying *SNHG1* could competitively bind to *hnRNPL* leading to downregulating of E-cadherin. **P < 0.01, ***P < 0.001.

To explore whether *SNHG1* exerts its biological function by binding to *hnRNPL*, a rescue assay between *SNHG1* and *hnRNPL* was performed. Transwell and wound healing assays revealed that *hnRNPL* knockdown significantly reversed the *SNHG1* overexpression-induced cell migration ability in PCa cells (Fig. 6A, B). Meanwhile, western blot showed that forced expression of *hnRNPL* reversed the *SNHG1* upregulating-induced inhibition of E-cadherin protein (Fig. 6C). Moreover, Survival analysis revealed that PCa patients with both low *SNHG1* and *HnRNPL* expression levels had better overall survival and disease-free survival with GEPIA (Fig. 6D). In general, we illustrated that LncRNA *SNHG1* contributed to PCa growth and metastasis by directly binding to *hnRNPL* to coregulate E-cadherin, which activates PCa EMT (Fig. 6E).

## Discussion

In recent years, the incidence of prostate cancer has been on the rise in China. Despite that the diagnosis rate of prostate cancer has been improved by diagnostic methods such as screening PSA and imaging, the mortality caused by PCa remains unchanged, and metastasis is a critical factor leading to the death of prostate cancer^15,16^. Therefore, to reveal the pathogenesis of PCa may help us find specific biomarkers, which bears great significance for the diagnosis, treatment and prognosis of patients with PCa.

LncRNAs participates in the development of cancer through a variety of mechanisms that regulate transcription, translation and protein function^17^. LncRNA prostate cancer antigen 3 (PCA3) has been approved by the U.S. FDA as a marker for the diagnosis of PCa, and has also been included in “Guidelines for Diagnosis and Treatment of Urological Diseases in China”^18^, announcing tremendous potential of lncRNA as a biomarker for the diagnosis and treatment of PCa, which is worthy of in-depth study. Although several studies have proved that *SNHG1* promoted PCa proliferation via serving as “miRNA sponeges”^11^^,^^12^, the functions and potential mechanisms in PCa remains unclear. We identified lncRNA *SNHG1* was overexpressed in prostate cancer tissues and cells, and associated with patient’s clinical characteristics. Results of gain and loss of function shown that lncRNA *SNHG1* promoted PCa cell proliferation and migration in vitro, and loss-of *SNHG1* inhibited vivo tumor growth, suggesting that *SNHG1* is essential in PCa progression.

It is well known that LncRNAs mainly act as ceRNA to backout the inhibiting effects of miRNAs on their downstream targets^19,20^. *SNHG1* was also reported to regulate miR-199a-3p and miR-337-3p/AKT2 axis, leading to promotion in PCa cells^11,12^. However, recently studies revealed that RNA binding protein plays a crucial role in LncRNA regulating network. Peng et al. revealed that Lnc-FAM84B-4 interacts with protein hnRNPK to restrain MAPK pathway, leading to CRC progression^21^. Klingenberg et al. indicated that CASC9/*hnRNPL* serves as a lncRNA/protein complex associated with clinically relevant viability and affects AKT signaling in HCC^22^. Therefore, RNA-Pulldown and RIP was constructed to establish the *SNHG1* and *hnRNPL* interaction in PCa cells. *hnRNPL* belongs to the hnRNP protein family, which participates in the malignant progression of tumors though mediating alternative splicing of precursor mRNA, strengthening mRNA stability, regulating mRNA translation etc^23–26^. Linked to our results, we demonstrated that the binding of *SNHG1* to *hnRNPL* is responsible for a part of its cancer-promoting features in PCa.

The EMT is a dynamic and critical process that enable the transition from early cancer to aggressive cancer, characterized via a decrease of epithelial markers and a upregulation in mesenchymal markers and EMT-related TFs^27^, but the upper mechanisms regulating EMT require fully understanding. The major epigenetic events, such as DNA methylation, histone modification, nucleosome-reshaping, and noncoding RNAs, play vital roles in activation or suppression of the initiation and sustainability of EMT^28–30^. CDH1, also known as E-cadherin, Notably, loss of E-cadherin was reckoned as the crucial step to initiate EMT that sustained PCa metastasis. Our works exhibited that the interaction of *SNHG1* with *hnRNPL* is responsible for a part of EMT through next generation sequencing and RIP assays in PCa cells. Furthermore, the *SNHG1*/*hnRNPL* complex lead to a downstream of E-cadherin via binding to its mRNA, which promotes EMT in prostate cancer. These results also provide a new sight in *SNHG1* regulating network.

Based on previous observations, we provide a strong evidence that *SNHG1* interacts with RNA-binding protein *hnRNPL* to activate EMT pathway, promoting the malignant progression of PCa, which is expected to provide a comprehensive understanding of the way *SNHG1* acts on cancers. This thorough comprehension of the interaction relationship of *SNHG1* and *hnRNPL* may lead to more valid strategies to diagnose and treat PCa.

## Conclusion

In conclusion, we found that *SNHG1* promotes the proliferation and progression of PCa, and high expression of both *SNHG1* and *hnRNPL* can shorten the overall and disease-free survival of PCa patients. Further experiments demonstrated that *SNHG1* can interact with *hnRNPL* and ultimately leads to down-regulation of E-cadherin expression and promote EMT progression, leading to tumor metastasis in PCa.

## Materials and methods

### Patients and clinical samples

Formalin fixed paraffin—embedded prostate cancer specimens were obtained from patients that undergo radical prostatectomy (RP) at zhujiang hospital from 2015 to 2018. The pathological types of the control group (total 14 specimens) were benign prostatic hyperplasia (BPH) or adjacent prostatic tissues. Patients’ clinical information was obtained by reviewing the follow-up records of their electronic medical records. The median age of the enrolled patients was 67.5 years and average age was 63.3 (range: 22–82 years). Clinical TNM staging and Gleason scores of patient specimens were based on the American Joint Committee on Cancer Eighth Edition (2017) and the 2016 World Health Organization classification of genitourinary tumors. All patients agreed to participate in this study by signing an informed consent in accordance with the ethical scheme formulated by the ethics committee of Zhujiang Hospital, Southern Medical University. We were blinding to the groups when assessing the outcome.

### Survival analysis

Association of LncRNA *SNHG1* and *hnRNPL* level on clinical events was evaluated using The Cancer Genome Atlas (TCGA) prostate adenocarcinoma cohort. Survival and expression data were acquired from TCGA data portal. Gene Expression Profiling Interactive Analysis (GEPIA) was utilized to analyze Overall survival and disease-free survival. Statistical significance was evaluated by Kaplan–Meier analysis and log-rank test.

### Fluorescence in situ hybridization (FISH)

Fluorescence in situ hybridization was constructed to identify the intracellular expression and sublocalization of *SNHG1* according to the protocol described before^31^, with minor modifications. PCa cells were grown on 15 mm confocal dishes (JET BIOFIL, Guangzhou, China). Probe sequences targeting homo-*SNHG1* were designed and generated by Ribo Bio (Guangzhou, China). Positive control probe sequences targeting homo-18S (cy3, Ribo Bio, Guangzhou, China), All operations were carried out in accordance with the Ribo™ Fluorescent In Situ Hybridization kit instructions, and minor modifications were made during the prostate tissue FISH assay. The samples were captured in Olympus laser scanning microscope FV3000 (Olympus Corporation, Japan). Images were processed in ImageJ through 3 major steps: background subtraction, set a threshold to label all the cells and label the *SNHG1* positive cells.

### Cytoplasmic and nuclear RNA isolation

Cytoplasmic and nuclear RNA was extracted using a Nuclear/Cytoplasmic Isolation reagents (No. 78833, Thermo Fisher Scientific, United States) according to the manufacturer’s instructions. And qRT-PCR was applied for further detection.

### Cell culture and cell lines

We used five human prostate cancer cell lines and one immortalized prostatic epithelial cell line, all the cells including RWPE-1, LNCaP, 22Rv1, PC-3, DU145 were obtained from Stem Cell Bank, Chinese Academy of Sciences. RPMI-1640 medium containing 10% fetal bovine serum (FBS, Hyclone) was used to culture all prostate cancer cells, and the Keratinocyte Serum Free Medium (KSFM) (Gibco, No. 10744-019) added in 5 ng/mL epidermal growth factor (EGF) (Gibco, No. 10450-013) was suitable for culturing RWPE-1 cells. A Wild environment (37°C in 5% CO_2_) was set for maintaining all cells.

### Construction of cells with stable knockdown of *SNHG1*

We designed and synthesized three small—interfering RNAs targeting *SNHG1* (NR_003098.2) and negative control (NC) siRNA negative control sequence without a specific target, all the siRNAs were synthesized by GeneChem (Shanghai, China). The sequence of siRNAs targeting *SNHG1* is as follows, si-h-*SNHG1*_001: CAGCA GTTGA GGGTT TGCTG TGTAT; si-h-*SNHG1*_002: TTCAA CAGCT AGGTT GTCCTT; si-h-*SNHG1*_003: GACCU AGCUU GUUGC CAAUTT. *SNHG1* overexpressed cells used pcDNA3.1 system, which was synthesized by VigeneBio co. (Shandong, China). In this study, we used lentiviral vectors (GeneChem, Shanghai, People’s Republic of China) to construct stable silencing *SNHG1* PCa cell lines following to the authoritative manufacturer’s instruction. Target sequence of shRNA (short hairpin RNA) againsting *SNHG1* was 5’-GACCU AGCUU GUUGC CAAU-3’. Stable *SNHG1* silenced cell lines sh-*SNHG1* were kept in 5 μg/mL puromycin for 10 days and then confirmed that it is validity and specificity.

### Quantitative RT–PCR assay and RNA extraction

The RNAiso Plus reagent (TaKaRa) and PrimeScript RT reagent Kit (TaKaRa) were utilized to extract total RNA and generate cDNA according to the official protocols. Extraction of nuclear and cytoplasmic RNA utilized the Ambion® PARIS™ system according to the kit procedures. The quantitative reverse transcriptase–PCR (qRT–PCR) reactions was performed with the SYBR Green PCR Master Mix (TaKaRa) with ABI7500 Fast Real-Time RCR System (Applied Biosystems, USA). The Primer3 system was utilized to design the gene unique primers which were subsequently synthesized through TsingKe Biological Technology (Guangzhou, China). Every survey was conducted in three times, and GAPDH was utilized to normalize the results as internal reference. The relative quantification method which calculated by 2^-ΔΔCt^ was used in analyzing the expression data.

### CCK-8 assay

2,000 PCa cells were seeded per well in 96-well plates, and Each well contained medium containing 10% FBS with a total volume of 100 ml. According to the counting kit-8 (CK-04, Dojindo) manufacturer’s operating instructions, after the cells were adherent to the plates, the original medium was removed, RPMI1640 diluted CCK-8 reagent (10% v/v) in 100 μl was added to the cells, following 2 h of incubated. Then, the optical density (OD) at 450 nm was measured by a microplate reader (EXL800, BioTek Instruments). Experiments were performed in triplicate.

### Colony formation

PCa cells were inoculated into 6 pore plates at a rate of 500 cells per well in a 2 mL medium containing 10% fetal bovine serum, and incubated for two weeks for colony formation analysis. Then, 4% paraformaldehyde were used to fix the colonies and Giemsa was applied to stain colonies. The experiment of colony formation was repeated three times with three Wells in each group.

### EdU incorporation assay

Cell-Light EdU staining kit (RiboBio, Guangzhou, China) was utilized to detect cell proliferation activity according to the manufacturer’s instructions. Images were obtained by a fluorescence microscope (Olympus, Tokyo, Japan) at 200×. The proportion of EdU positively stained cells to Hoechst-stained cells (with blue fluorescence) in per well was equal to the cell proliferation rate. Experiments were performed in triplicate.

### Transwell migration assay

Transwell (Costar, Corning, USA) with a membrane of numerous pole(8.0μm) was used to perform Cell migration assay. 35000 cells were hybridized in FBS-free RPMI-1640 and sowed into the upper chamber of the well. Then, 500 μL complete medium with FBS was put into the sublayer. After 24 h, 4% paraformaldehyde and Giemsa (Boster Ltd., Wuhan, China) were used to fix and stain the cells. After that, wiping out the cells on the top surface of the membrane, and the cells on the bottom surface were photographed through an inverted microscope. (Olympus DP72). Five randomly visual fields were chosen to count the cell number by using Image J software. Experiments were performed in triplicate.

### Mice xenograft analysis

Xenograft models were randomly created through persistent injection of 5 × 10^6^ DU145/sh-NC, DU145/sh-*SNHG1* cells (*n* = 7 per group), on the axillae of nude male mice (4–5 weeks). Tumor sizes were measured twice a week and formula $\frac{{\pi \times {\mathrm{Length}} \times {\mathrm{Width}}^2}}{6}$ was used to calculate tumor volume. Mice were raised in Specific Pathogen Free (SPF) environments for 4 weeks. Subsequently, tumor samples were carefully resected, photographed and specimens were further investigated by hematoxylin and eosin(H&E) and IHC. BALB/c nude male mice were purchased from the Animal Center of Southern Medical University, Guangzhou, China. The Animal Care and Use Committee of Southern Medical University approved all our experimental animal programs.

### Antibodies and western blot analyses

Related cell lysates were run on 4–12% SDS PAGEs to conduct Western blot analyses. RIPA lysis buffer containing protease inhibitors (#KGP250, KeyGEN BioTECH, Nanjing, China) was used to extracted PCa cells protein following to the operation protocol. Then, equal amounts of 30 μg proteins were separated by SDS/PAGEs and transferred onto PVDF membranes (Millipore, Billerica, MA, USA) electrically. Then, membranes were blocked with TBS(Tris/saline solution with 0.1% Tween-20) including 5% milk without fat for 1 h and incubated overnight at 4 °C covering by unique antibodies: rabbit anti-β-actin (#4970, CST), rabbit anti-Slug (#9585), rabbit anti-ZEB1(#3396, CST), rabbit anti-E-Cadherin (#3195, CST) and rabbit anti-*hnRNPL* (4D11, #ab6106, Abcam). All membranes were subsequently incubated at room temperature for 1 h with horseradish peroxidase-linked secondary anti-rabbit or anti-mouse IgG antibodies (Cell Signaling Technology). Final bands were visualized by ECL kit (Pierce Biotechnology, Rockford, IL, USA). Image J was applied to quantify the intensity of the band. Experiments were performed in triplicate.

### Plasmid transfection

The full-length wild-type human *SNHG1* and 401-607 bp truncated mutant *SNHG1*-mut were inserted into pcDNA3.1 expression vector acquired from Shangdong Vigene Biosciences Co. All constructs were confirmed by sequencing. Referring to the Lipofectamine™ 3000 Transfection Reagent instructions (L3000015, Thermo Fisher Scientific, USA), DU145/sh-*SNHG1* cell line was transiently transfected with *SNHG1*-mut or *SNHG1*, DU145 and C4-2 cells were transfected with *SNHG1* or meaningless vector. Expression of Plasmid was confirmed by qRT-PCR.

### Immunohistochemical analysis and assessment

Prostate tissue IHC were employed to investigate protein expression in tissues as described previously. Relative proteins expression was investigated in Prostate tissue IHC as previously referred to^32^. In IHC, we incubated the specimens with 1: 500 anti-Ki-67 antibodies. IHC results were evaluated by calculating the percentage of positive nuclei, the percentage of positive cells and the staining concentration. Every assessment was carried out by three separated advanced pathologists utilizing the parallel microscope. Experiments were performed in triplicate.

### RNA-seq processing

RNA sequencing and sequence quality control was performed by BGISEQ platform. Human genome reference was established from UCSC version GRCh38/hg38 chromosomes 1-22, X, Y and mitochondrial DNA. The further analysis including heatmap, gene set enrichment analysis, and gene annotation of sequencing data was completed by BGI Dr. Tom system.

### RNA-protein pulldown and RNA immunoprecipitation (RIP)

In vitro biotin-labeled RNAs (*SNHG1*, AR-3’UTR and Ploy (A)_25_) and RNA-protein pull-down assay were done with Pierce™ Magnetic RNA-Protein Pull-Down Kit (#20164, Thermo Scientific™). Pull-down protein samples were ran on SDS-PAGE gels, following silver staining to Identify the difference bands, then the difference bands were analyzed by mass spectrometry. RIP assays were performed by utilizing EZ-Magna RIP™ Kit (#17-701, Merck Millipore) following the protocol.

### In silico binding prediction

To obtain potential *SNHG1* and *hnRNPL* binding sites, we first predicted the interaction probability between *SNHG1* and *hnRNPL* sequences in RNA-Protein Interaction Prediction(RPISeq). Furthermore, catRAPID was used to predict the binding site of *hnRNPL* on *SNHG1*. The highest-raking site at RNA position 401-677 bp includes CA-rich motifs and thus used for subsequent analysis.

### Statistical analysis

Statistical analyses were carried out with GraphPad Prism8 (GraphPad Software, La Jolla, USA). Student’s *t* test or chi-square test was applied to determine the statistical significance of differences between distinct groups, if proper. All results were expressed as mean ± standard deviation (SD), and the two-tailed *p* value of 0.05 was considered statistically significant.

### Supplementary information

Supplementary Figure Legends

Figure S1

Potential interactors of SNHG1

## Data Availability

All data generated or analyzed during this study are included in this published article.
