# Correction: LncRNA *SNHG1* and RNA binding protein *hnRNPL* form a complex and coregulate *CDH1* to boost the growth and metastasis of prostate cancer

**DOI:** 10.1038/s41419-024-06561-5

**Published:** 2024-03-14

**Authors:** Xiao Tan, Wen-bin Chen, Dao-jun Lv, Tao-wei Yang, Kai-hui Wu, Li-bin Zou, Junqi Luo, Xu-min Zhou, Guo-chang Liu, Fang-peng Shu, Xiang-ming Mao

**Affiliations:** 1 Department of Urology, The Affiliated Hospital of Southwest Medical University, Luzhou, Sichuan China; 2 Department of Urology, Zhujiang Hospital, Southern Medical University, Guangzhou, Guangdong China; 3 Department of Urology, Minimally Invasive Surgery Center, the First Affiliated Hospital of Guangzhou Medical University, Guangdong Key Laboratory of Urology, Guangzhou Institute of Urology, Guangzhou, Guangdong China; 4 Department of Urology, Guangzhou Women and Children’s Medical Center, Guangzhou Medical University, Guangzhou, Guangdong China

**Keywords:** Metastasis, Prostate cancer

Correction to: *Cell Death and Disease* 10.1038/s41419-021-03413-4, published online 01 February 2021

In this article Fig. 4 has been updated:
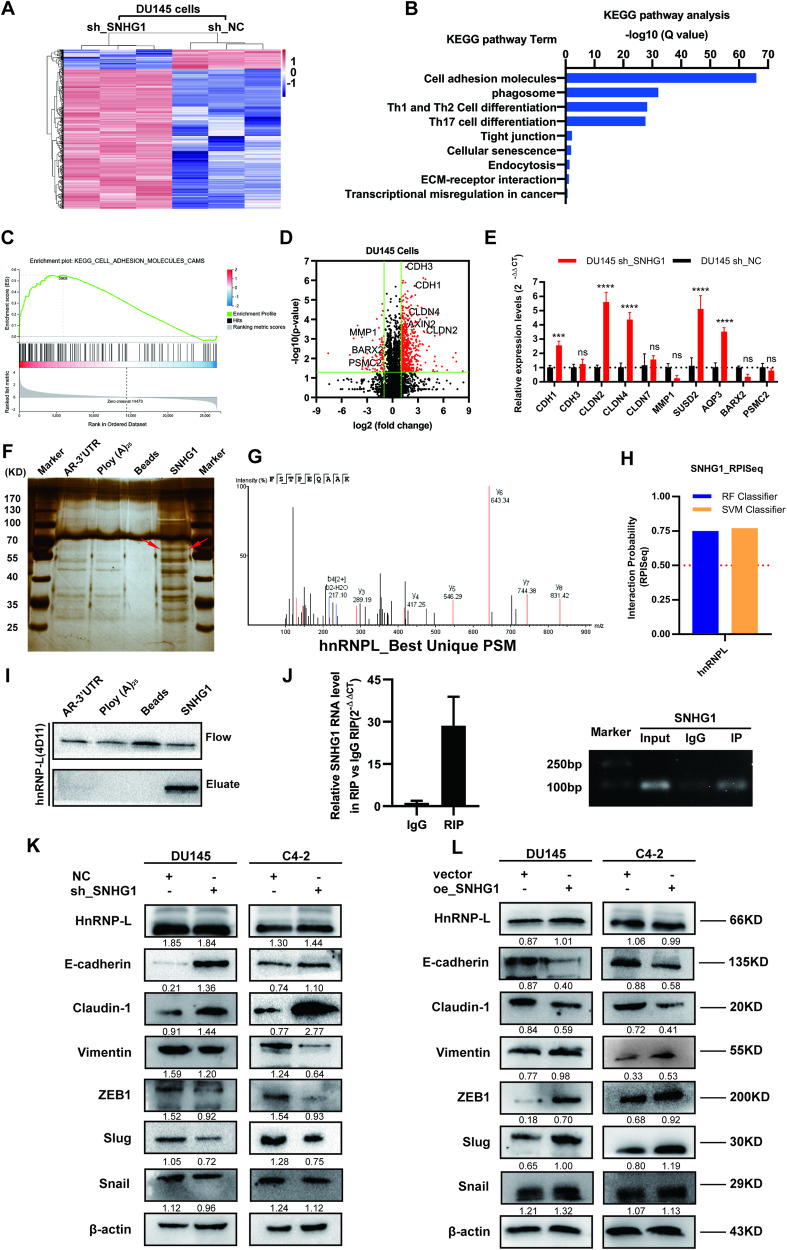

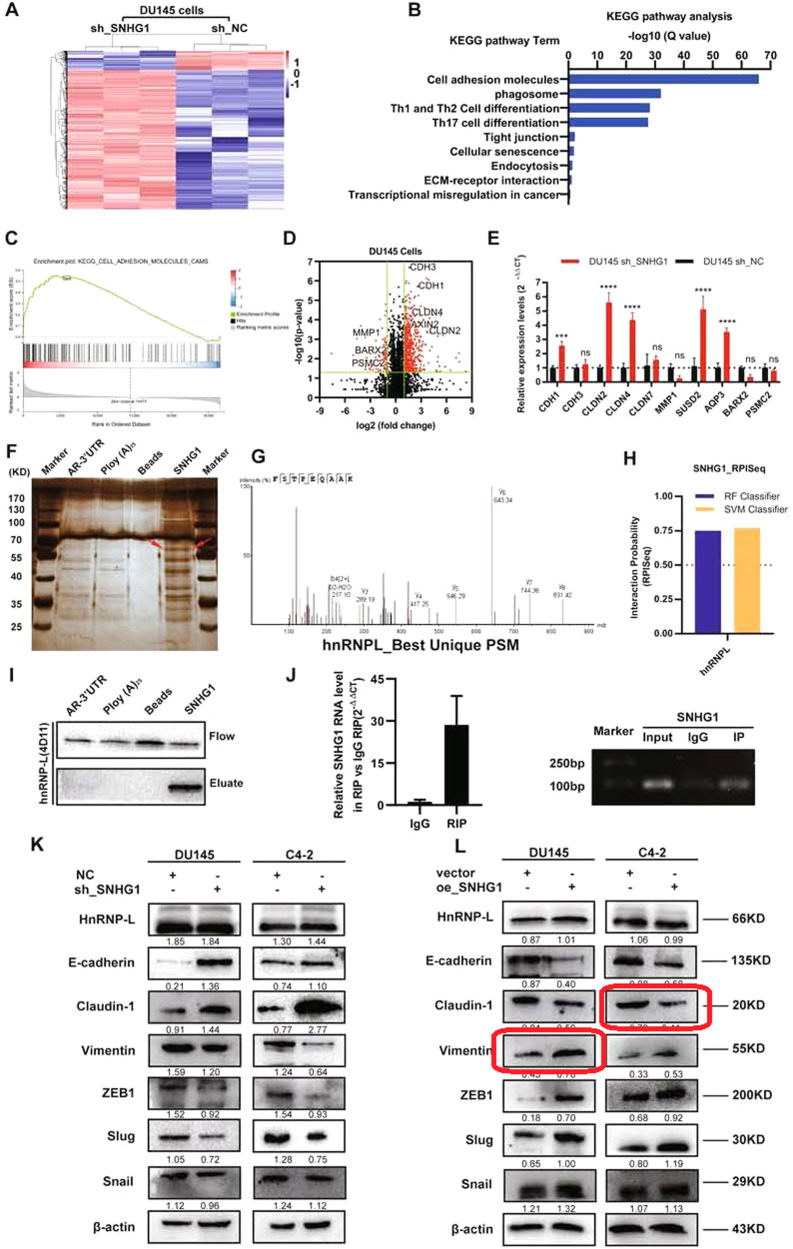


Fig. 4: SNHG1 could directly bind to hnRNPL and regulate EMT.

**A** Hierarchical clustering of 1045 transcripts altered (≥1.5-fold change) in NC-treated cells and shRNA SNHG1-treated cells with three repeats. **B**, **C** KEGG analysis and differential genes GSEA enrichment analysis demonstrated that cell adhesion molecules are the potential targets of SNHG1 pathway. **D** Volcanic map analysis of differential genes. **E** The altered mRNA levels of genes were selectively confirmed by qRT-PCR in knockdown SNHG1. **F** SDS-PAGE silver staining of RNA pull-down protein samples showed a significant difference in protein band from 55 to 70 kDa. **G** Differential protein band mass spectrometry showed that the protein was hnRNPL. **H** The interaction possibilities of hnRNPL and SNHG1 were detected in RPIseq, and the results showed that hnRNPL could well bind with SNHG1 well (RPISeq). **I** Pull-down assays showed that biotinylated SNHG1 could retrieve hnRNPL in DU145 cells by western blot(flow-through was a input control). **J** RNA immunoprecipitation revealed that hnRNPL could also specifically bind to SNHG1. **K**, **L** The western blotting results after SNHG1 knockdown in DU145 and C4-2 cells showed that the expression level of hnRNPL did not change, while E-cad and Claudin-1 were significantly up-regulated, Vimentin, Slug and ZEB1 were significantly decreased. overexpression of SNHG1 showed an opposite effect of these protein in in DU145 and C4-2 cells. Error bars indicate means ± SD. ***P < 0.001, ****P < 0.0001.

The original article has been corrected.

