# Supplementary material for: LncRNA SNHG1 and RNA binding protein hnRNPL form a complex and coregulate CDH1 to boost the growth and metastasis of prostate cancer
Source: Cell Death Dis. 2021 Feb 1;12(2):138. doi: 10.1038/s41419-021-03413-4 (PMC7862296; doi:10.1038/s41419-021-03413-4)
Supplement: Supplementary file 1 — Supplementary Figure Legends [file 41419_2021_3413_MOESM1_ESM.docx]

**Figure S1.** **LncRNA *SNHG1* could not promote RWPE-1 cells migration abilities in vitro. (A)** Relative RNA expression levels of SNHG1 after silencing or overexpressing SNHG1 in DU145 and C4-2 cells. **(B)**Relative RNA expression levels of SNHG1, E-cadherin mRNA, and Vimentin mRNA after overexpressing SNHG1 in RWPE-1cells. **(C and D)** Transwell assays and would healing assays showed that SNHG1 overexpression did not promote RWPE-1 cell migration. **(E)** Relative protein expression levels of E-cadherin and Vimentin after SNHG1 overexpressed in RWPE-1 cells. GAPDH as the internal reference. **P<0.01.
