# Supplementary figures and images for: LncRNA SNHG1 and RNA binding protein hnRNPL form a complex and coregulate CDH1 to boost the growth and metastasis of prostate cancer
Source: Cell Death Dis. 2021 Feb 1;12(2):138. doi: 10.1038/s41419-021-03413-4 (PMC7862296; doi:10.1038/s41419-021-03413-4)

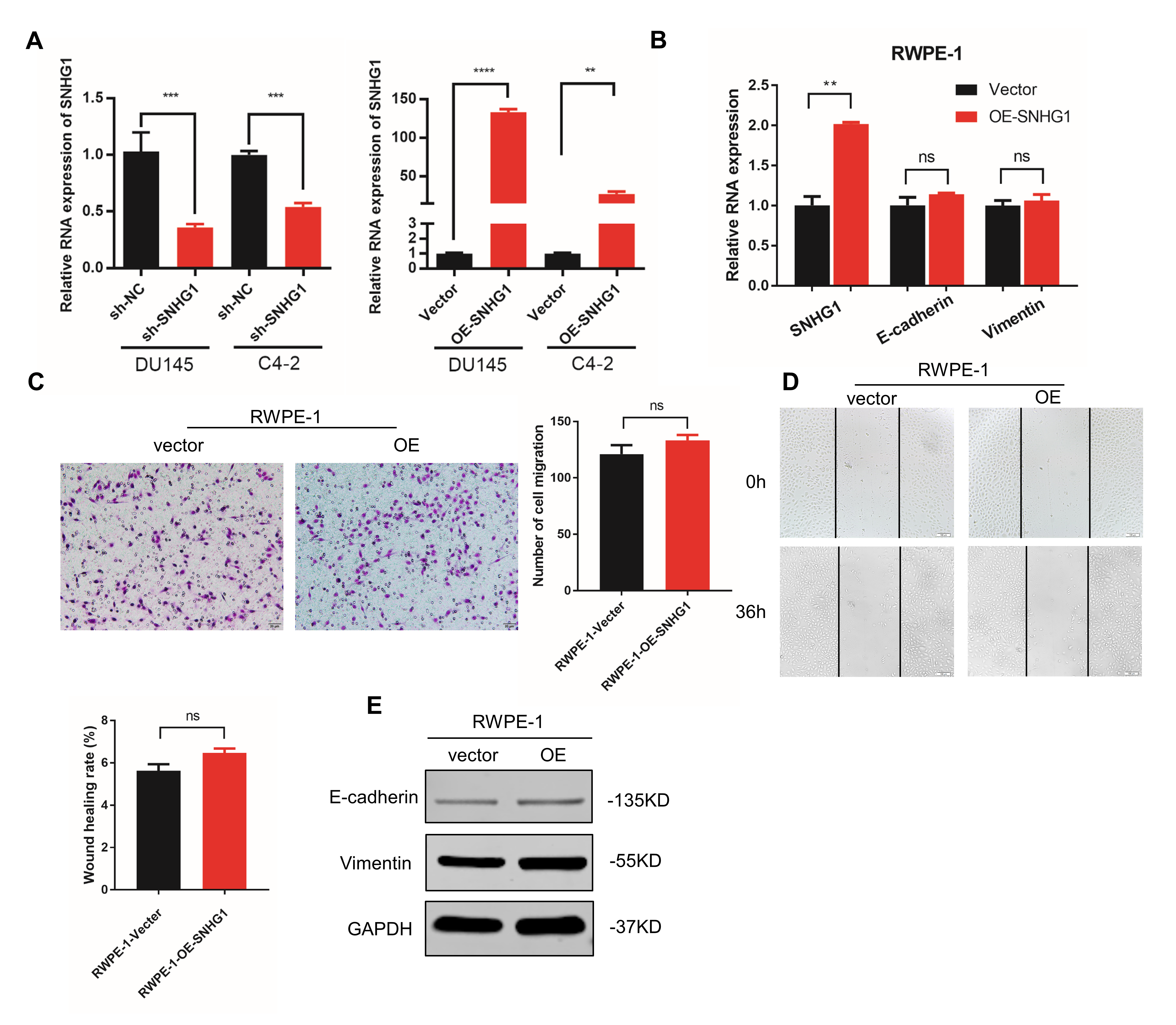

Supplement: Supplementary file 2 — Figure S1 [file 41419_2021_3413_MOESM2_ESM.tif]
